# Low total osteocalcin levels are associated with all-cause and cardiovascular mortality among patients with type 2 diabetes: a real-world study

**DOI:** 10.1186/s12933-022-01539-z

**Published:** 2022-06-09

**Authors:** Yun Shen, Lei Chen, Jian Zhou, Chunfang Wang, Fei Gao, Wei Zhu, Gang Hu, Xiaojing Ma, Han Xia, Yuqian Bao

**Affiliations:** 1 Department of Endocrinology and Metabolism, Shanghai Clinical Center for Diabetes, Shanghai Key Clinical Center for Metabolic Disease, Shanghai Diabetes Institute, Shanghai Key Laboratory of Diabetes Mellitus, Shanghai Jiao Tong University Affiliated Sixth People’s Hospital, 600 Yishan Road, Shanghai, 200233 China; 2 Division of Vital Statistics, Institute of Health Information, Shanghai Municipal Center for Disease Control and Prevention, 1380 West Zhongshan Road, Shanghai, 200336 China; 3 Chronic Disease Epidemiology, Pennington Biomedical Research Center, Baton Rouge, LA 70808 USA

**Keywords:** Osteocalcin, All-cause mortality, CVD mortality

## Abstract

**Background:**

The association between osteocalcin and mortality has been scantly studied. We aimed to investigate the association between osteocalcin along with its trajectories and mortality based on long-term longitudinal data.

**Methods:**

We performed a retrospective cohort study of 9413 type 2 diabetic patients with at least three measurements of total serum osteocalcin within 3 years since their first inpatient diagnosis of type 2 diabetes. Baseline, mean values of osteocalcin levels and their trajectories were used as exposures. A multivariable-adjusted Cox proportional hazards model was used to estimate the association of osteocalcin levels and their trajectories with mortality.

**Results:**

During a mean follow-up of 5.37 years, 1638 patients died, of whom 588 were due to cardiovascular events. Multivariable-adjusted hazard ratios (HRs) across quintiles of baseline osteocalcin levels were 2.88 (95% confidence interval (CI) 2.42–3.42), 1.65 (95% CI 1.37–1.99), 1.17 (95% CI 0.96–1.42), 1.00, and 1.92 (95% CI 1.60–2.30) for all-cause mortality, and 3.52 (95% CI 2.63–4.71), 2.00 (95% CI 1.46–2.73), 1.03 (95% CI 0.72–1.47), 1.00, 1.67 (95% CI 1.21–2.31) for CVD mortality, respectively. When we used the mean values of osteocalcin as the exposure, U-shaped associations were also found. These U-shaped associations were consistent among patients of different baseline characteristics. Patients with a stable or even increasing trajectory of osteocalcin may have a lower risk of both all-cause and CVD mortality.

**Conclusions:**

A U-shape association between baseline osteocalcin and mortality was observed among patients with type 2 diabetes. Patients with lower levels of serum osteocalcin during follow-ups had higher risks for all-cause and cardiovascular mortality.

**Supplementary Information:**

The online version contains supplementary material available at 10.1186/s12933-022-01539-z.

## Background

The crosstalk between bone turnover and metabolic disease has been long discussed during the past decades. Bone has been considered an endocrine organ that is fully involved in energy and metabolism [1]. Osteocalcin, a bone-derived hormone, is mainly secreted by osteoblasts. It can regulate glycol-lipid metabolism and mediates the interaction between bone metabolism and cardiovascular diseases [2]. We have conducted a series of studies in terms of osteocalcin and metabolic diseases. In population studies, our previous findings indicated that there was a close relationship between osteocalcin and glucose levels [3], lipid profiles [3] as well as body fat distribution [4–6]. We also found that osteocalcin was well associated with the incidence of type 2 diabetes [7], non-alcoholic fatty liver disease [8–10], and metabolic syndrome [11, 12]. Multiple metabolic disorders would finally contribute to the aggregation of cardiovascular phenotypes and the causality would inevitably be continued. Long-term follow-up of our series of studies indicated that osteocalcin was associated with adverse cardiovascular events including heart failure, nonfatal myocardial infarction, repeat coronary revascularization, hospitalization for unstable angina, and nonfatal stroke [13, 14]. For basic science, our previous study demonstrated that osteocalcin has an endothelial-protective effect on atherosclerosis mediating the PI3K/Akt/eNOS signaling pathway [15]. Therefore, the evidence linking osteocalcin and metabolism was convincing either based on population or basic studies. However, the association between osteocalcin and hard endpoint events (i.e. mortality) is rarely studied and the conclusions are inconsistent, especially in patients with type 2 diabetes. In addition, the findings from population studies by our team were mostly based on cross-sectional studies and a single measurement of osteocalcin was used in these studies. The changes and trajectories of osteocalcin were less studied based on long-term longitudinal data.

Evidence from selected population cohorts is somehow limited by the sample size and low incidence of endpoint events. To fill in these gaps, longitudinal real-world data from electronic health records with high quality are better for the population hypothesis. Shanghai Clinical Center for Diabetes under the supervision of Shanghai Sixth People’s Hospital was established in 2002 and electronic health records (EHRs) have been widely used since 2000. In the past 20 years, more than 100 thousand patients with diabetes have received healthcare services in the Shanghai Clinical Center for Diabetes and were registered during regular follow-ups. Therefore, in this EHR-based study, we aimed to investigate the association between osteocalcin and the risks of all-cause and cardiovascular disease (CVD) mortality among patients with type 2 diabetes.

## Methods

### Study design and participants

The Shanghai Clinical Center for Diabetes was established in 2002 and more than 100,000 patients with diabetes including type 1 diabetes, type 2 diabetes, gestational diabetes, and other specific types of diabetes have received healthcare services in the past 20 years. For those patients of permanent residents in Shanghai and other patients who were willing, a registered archive was established and healthcare data including anthropometric, demographic, laboratory, and diagnostic characteristics were collected during the first inpatient visit. Subsequent follow-up visits were performed according to the recommendations in the guideline for the prevention and treatment of type 2 diabetes in China (updated every 3–4 years). This was an observational dynamic cohort study and we obtained written approval from the Institutional Review Boards of Shanghai Jiao Tong University Affiliated Sixth People’s Hospital. We did not obtain informed consent from participants involved in our study because we used anonymized data compiled from EHRs.

**Fig. 1:**
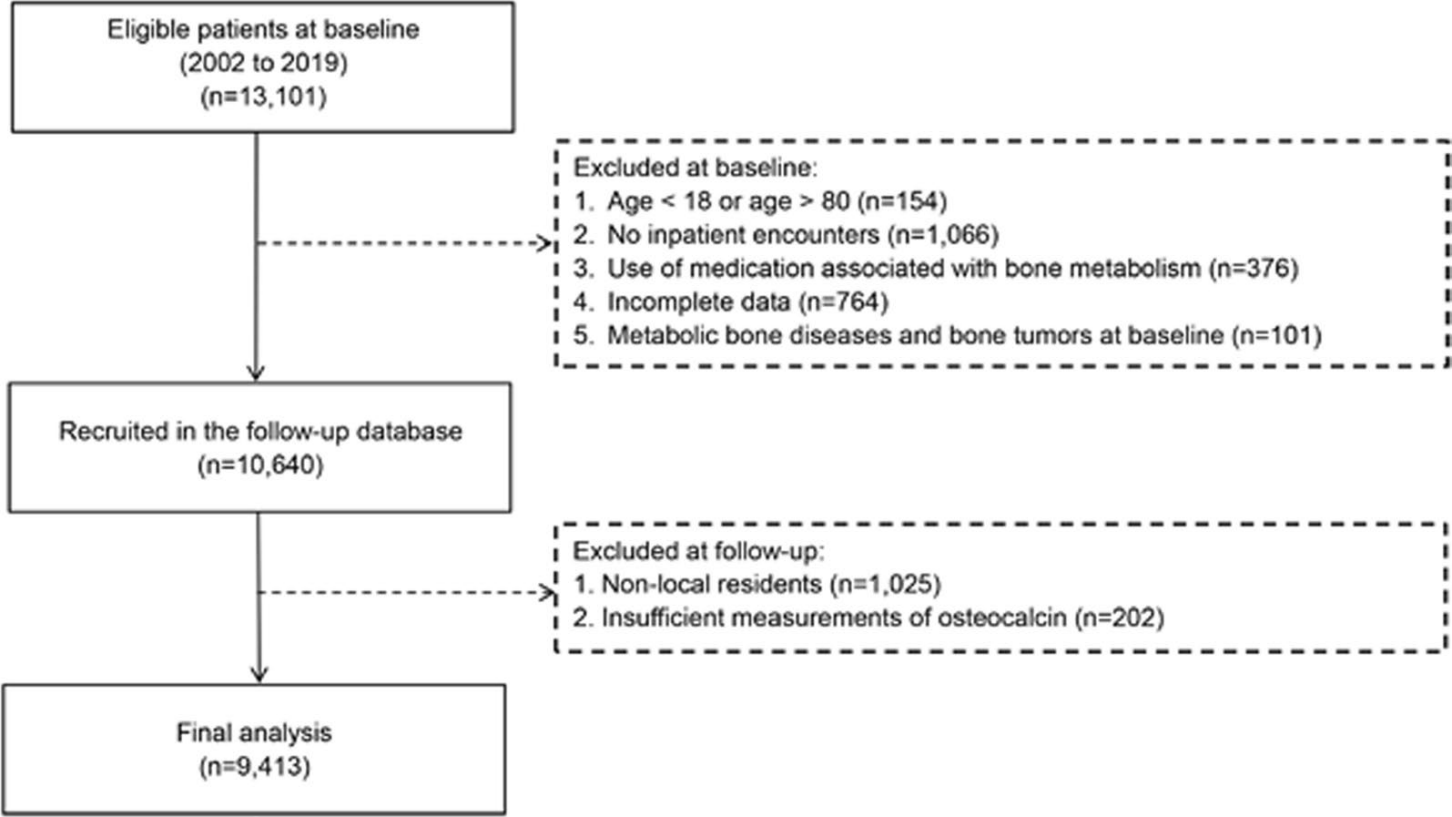
Flow chart of the study

Patients with type 2 diabetes, who were on regular visits with at least three measurements of osteocalcin within 3 years since their first date of inpatient visit according to the data of archive, were eligible for this study. Additional inclusion criteria were: (1) patients aged from 18 to 80 years old; (2) patients without the use of any medications that were associated with the bone metabolism; (3) patients with complete data on exposure and covariates. To avoid the bias of reverse causality, patients with metabolic bone diseases and bone tumors at baseline were excluded. The final samples for analysis included 9413 patients with type 2 diabetes (see flow chart in Fig. 1).

### Measurements

We created a follow-up database in electronic form using the unique personal identification number (issued by the government). Patients’ information on the date of birth, sex, age of diabetes diagnosis, smoking status, and the use of medication prescriptions such as antihypertensive drugs, glucose-lowering drugs, lipid-lowering drugs, and use of antiplatelet or anticoagulant drugs was collected through a standardized electronic inpatient medical record data collection form. Moreover, patients’ height, weight, blood pressure, total cholesterol, triglyceride, high-density lipoprotein (HDL) cholesterol, low-density lipoprotein (LDL) cholesterol, hemoglobin A1c (HbA1c), serum creatinine, serum osteocalcin, and 25 hydroxyvitamin D (25(OH)D) were measured through standardized methods at each inpatient admission. Body mass index (BMI) was calculated as weight (kg)/height^2^ (m^2^). The estimated Glomerular Filtration Rate (eGFR) was estimated using the Chronic Kidney Disease Epidemiology Collaboration equation (CKD-EPI) formula [16].

Serum total osteocalcin (hereinafter referred to as serum osteocalcin) values were extracted from the database, which was quantified via an electro-chemiluminescent immunoassay (Roche Diagnostics GmbH, Mannheim, Germany) in the central laboratory of Shanghai Jiao Tong University Affiliated Sixth People’s Hospital as reported in our series of studies and the intra- and inter-assay coefficients of variation were 1.2–4.0% and 1.7–6.5%, respectively.

### Prospective follow-up and outcomes

Causes and time of death were obtained from the database of the Shanghai Municipal Center for Disease Control and Prevention and were linked with the study data through the personal identification number [17]. The death causes were identified with the use of the codes in the International Classification of Disease 10th version (ICD-10). The rate of missing death events in Shanghai was proven to be 0.7‰ (administrative data). We used chart review to evaluate the confirmation of death (COD) via the Shanghai adaptation of the Medical Record Audit Form. Trained physicians have reviewed the medical records of a death event and reassigned the COD, which provided a gold standard to measure the quality of routine COD data. The death events identified by Shanghai Civil Registration and Vital Statistics routine monitoring were thus reported with high sensitivity and specificity of 85.7% and 90.0%, respectively (personal communication). CVD mortality was defined according to causes of death by ICD-10 codes from I00–I99. In the current study, the primary outcome was all-cause mortality and the secondary outcome was CVD mortality. All patients were followed up until a death event occurred or until 31 December 2021, whichever occurred first. To avoid the potential immortal time bias, the baseline date was defined as the last date of osteocalcin measurement within 3 years following the first date of the inpatient visit due to diabetes.

### Statistical analysis

The primary analysis was to investigate the association between serum osteocalcin levels and all-cause and CVD mortality. Cox proportional hazards regression was used to estimate hazard ratios (HRs) for all-cause and CVD mortality according to quintiles of both baseline and mean levels of osteocalcin. Categories of osteocalcin levels were included in the models as dummy variables, and the significance of the linearity across categories of osteocalcin was tested in the same models by giving an ordinal numeric value for each dummy variable. The proportional hazards assumption in the Cox model was assessed with graphical methods and with models including time-by-covariate interactions. In general, all proportionality assumptions were appropriate. All analyses were first carried out adjusting for age and sex, and further for BMI, systolic blood pressure, HbA1c, LDL cholesterol, triglycerides, estimated GFR, 25(OH)D, smoking, use of antihypertensive drugs, use of glucose-lowering drugs, use of lipid-lowering drugs and use of antiplatelet or anticoagulant drugs. The trajectory analysis was considered the secondary analysis, which was performed using the latent class trajectory modeling [18]. A maximum likelihood approach was used to fit the model with the ‘hlme’ function from the ‘lcmm’ library in the R software environment. First, we scoped the model by the structure based on the clinical patterns in our dataset. This model was refined to confirm the optimal number of classes by the lowest Bayesian Information Criteria values. We then refine the optimal model structure from fixed through to unrestricted random effects of the model using the favored value derived in the last step. Next, we run model adequacy assessments including the posterior probability of assignments, odds of correct classification, and relative entropy. The mean trajectories with 95% predictive intervals for each class were finally plotted. Since serum osteocalcin values were abnormally distributed in this dataset, all the values including baseline, mean and trajectory were by log transformation before being fitted into the models. Statistical significance was considered to be *P* < 0.05. All statistical analyses were performed using R software 4.0.3 (R Foundation for Statistical Computing, Vienna, Austria).

## Results

The baseline characteristics are listed in Table 1. A total of 9413 patients (5519 men and 3894 women) with type 2 diabetes were analyzed with a mean age of 57.3 ± 14.2 years old. The median with an interquartile range of serum osteocalcin levels at baseline was 13.25 (10.61–17.70) ng/mL. Patients were divided into five groups according to the quintiles of baseline osteocalcin levels. As the baseline osteocalcin levels increased, the baseline age, duration of diabetes, BMI, blood pressure, cholesterol profiles, and HbA1c of the patients showed a linear trend across the quintiles (*P* for non-linearity > 0.05). However, other continuous variables such as triglycerides, vitamin D, and estimated GFR showed to be non-linear (*P* for non-linearity < 0.05). The glucose-lowering drugs across quintiles of baseline osteocalcin levels differed significantly. Most patients in Quintile 5 group were on insulin therapy (71.0%), metformin (88.9%), and α-glucosidase inhibitors (80.3%), while fewer were using insulin secretagogues (8.8%) and DPP4 inhibitors (8.3%).

**Table 1:** Baseline characteristics of patients with type 2 diabetes

	Baseline osteocalcin levels, ng/mL				
	Quintile 1	Quintile 2	Quintile 3	Quintile 4	Quintile 5
Participants (n)	1890	1879	1885	1878	1881
Age (years)	60.0 ± 13.4	57.4 ± 13.2	56.8 ± 13.7	56.7 ± 13.7	55.5 ± 16.5
Male (%)	64.6	65.6	59.4	54.4	49.2
Duration of diabetes (years)	7.21 ± 0.94	7.06 ± 0.96	7.01 ± 0.99	7.01 ± 1.01	6.87 ± 1.31
Body mass index (kg/m^2^)	24.5 ± 3.55	24.4 ± 3.51	24.2 ± 3.60	24.0 ± 3.59	23.6 ± 3.68
Blood pressure (mmHg)					
Systolic	128 ± 13	128 ± 15	128 ± 14	129 ± 15	130 ± 16
Diastolic	75 ± 13	73 ± 12	73 ± 12	74 ± 12	74 ± 12
Total cholesterol (mmol/L)	4.56 ± 1.23	4.62 ± 1.18	4.71 ± 1.17	4.74 ± 1.18	4.78 ± 1.33
Low-density lipoprotein cholesterol (mmol/L)	2.75 ± 0.94	2.77 ± 0.88	2.81 ± 0.91	2.85 ± 0.92	2.86 ± 1.02
High-density lipoprotein cholesterol (mmol/L)	1.11 ± 0.31	1.10 ± 0.31	1.10 ± 0.30	1.14 ± 0.32	1.14 ± 0.35
Triglycerides (mmol/L)	1.80 ± 0.95	1.77 ± 0.57	1.85 ± 0.85	1.66 ± 0.46	1.60 ± 0.29
HbA1c (%)	8.7 ± 2.0	8.6 ± 1.9	8.5 ± 2.0	8.4 ± 1.9	8.0 ± 2.0
25(OH)D (ng/mL)	16.0 ± 4.87	16.2 ± 4.86	15.7 ± 5.09	15.2 ± 5.21	14.4 ± 5.36
Estimated GFR (mL/min/1.73 m^2^) (%)					
≥ 90	65.2	69.9	68.8	68.9	57.3
60–89	31.8	27.4	28.8	28.9	34.7
< 60	3.0	2.7	2.4	2.2	8.0
Body mass index categories (%) (kg/m^2^)					
< 25	59.8	60.0	63.3	64.7	69.5
25–29.9	33.9	33.8	30.9	29.8	24.4
≥ 30	6.3	6.2	5.8	5.5	6.1
Current or past smoker (%)	28.3	34.5	31.4	25.3	20.6
Current drinker (%)	18.1	20.0	16.9	12.8	8.9
Family history of diabetes (%)	23.8	29.1	29.1	26.2	20.5
Insurance type (%)					
Insured	81.1	78.3	74.9	73.3	68.4
Uninsured	18.9	21.7	25.1	26.7	31.6
Use of medications (%)					
Antiplatelet or anticoagulant	15.2	19.6	16.6	12.3	14.9
Lipid-lowering	54.4	53.8	47.7	45.4	44.3
Antihypertensive	85.6	90.2	91.0	91.2	88.9
Glucose-lowering					
Metformin	74.9	84.7	86.7	86.4	88.9
Insulin	62.5	70.0	70.7	69.5	71.0
Insulin secretagogues	26.6	49.1	84.0	68.0	8.8
DPP4 inhibitors	92.2	74.3	26.5	19.8	8.3
α-glucosidase inhibitors	84.6	82.4	80.8	80.9	80.3
GLP-1 receptor agonists	2.1	9.0	16.4	10.1	0.7
Thiazolidinediones	44.2	52.0	51.5	51.2	51.1
SGLT2 inhibitors	2.2	3.0	2.6	2.1	2.6

### All-cause mortality

**Fig. 2:**
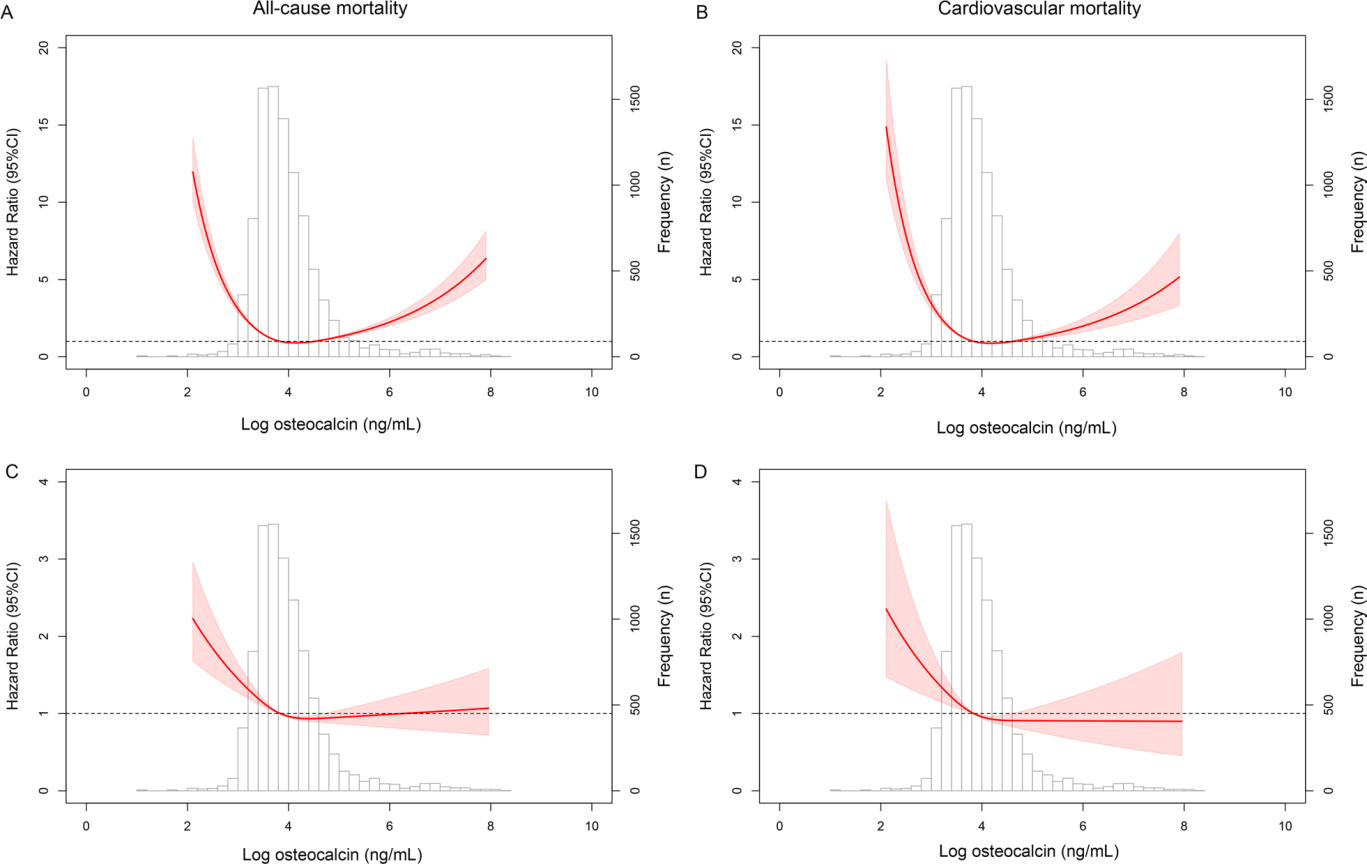
Restricted cubic spline curves for all-cause and CVD mortality. Serum osteocalcin levels were by log transformation before being fitted into the models. **A** and **B** Show multivariable-adjusted hazard ratios for all-cause and CVD mortality when baseline osteocalcin levels were applied. **C** and **D** Show multivariable-adjusted hazard ratios for all-cause and CVD mortality when mean osteocalcin levels were used. Variables adjusted included age, sex, body mass index, systolic blood pressure, HbA1c, low-density lipoprotein cholesterol, triglycerides, estimated GFR, 25(OH)D, smoking, use of antihypertensive drugs, use of glucose-lowering drugs, use of lipid-lowering drugs and use of antiplatelet or anticoagulant drugs

During a mean follow-up of 5.37 ± 2.50 years, 1638 patients died from any causes. Multivariable-adjusted HRs of all-cause mortality across quintiles of serum osteocalcin levels at baseline (Quintile 1 [< 10.24 ng/mL], Quintile 2 [10.24–12.08 ng/mL], Quintile 3 [12.08–14.68 ng/mL], Quintile 4 [14.68–19.19 ng/mL, set as reference group] and Quintile 5 [≥ 19.19 ng/mL]) were 2.88 (95% confidence interval (CI) 2.42–3.42), 1.65 (95% CI 1.37–1.99), 1.17 (95% CI 0.96–1.42), 1.00 and 1.92 (95% CI 1.60–2.30), respectively (Table 2). A significant U-shaped association with the risk of all-cause mortality was observed when serum osteocalcin at baseline was set as a continuous variable by using the restricted cubic spline curve (Fig. 2A). The lowest hazards occurred when baseline osteocalcin levels were considered as 14.22 ng/mL and 24.42 ng/mL, compromising the optimal range of serum osteocalcin levels at baseline in terms of the lowest risk of all-cause mortality from 14.22 to 24.42 ng/mL. When we used the updated mean values of serum osteocalcin as the exposures, the U-shaped association was slightly attenuated to an L-shaped curve (Fig. 2C). The corresponding HRs of all-cause mortality across quintiles of the updated mean of serum osteocalcin were 1.26 (95% CI 1.08–1.54), 1.22 (95% CI 1.01–1.46), 1.16 (95% CI 0.97–1.38), 1.00 and 1.07 (95% CI 0.91–1.27), respectively. The lowest hazards occurred when the mean osteocalcin level was considered as 14.32 ng/mL.

**Table 2:** Hazard ratios of mortality by different levels of osteocalcin at baseline and during follow-up among patients with type 2 diabetes

	Osteocalcin (ng/mL)				
	Quintile 1	Quintile 2	Quintile 3	Quintile 4	Quintile 5
All-cause mortality					
Baseline value					
No. of participants	1890	1879	1885	1878	1881
No. of cases	579	290	210	182	377
Person-years	9557	9813	10,359	10,489	10,320
Age- and sex-adjusted HR	2.68 (2.26–3.17)	1.59 (1.32–1.92)	1.15 (0.95–1.41)	1.00	2.19 (1.84–2.62)
Multivariable adjusted HR	2.88 (2.42–3.42)	1.65 (1.37–1.99)	1.17 (0.96–1.42)	1.00	1.92 (1.60–2.30)
Mean value					
No. of participants	1884	1883	1884	1882	1880
No. of cases	386	346	329	283	294
Person-years	9881	10,204	10,316	10,058	10,079
Age- and sex-adjusted HR	1.26 (1.06–1.50)	1.21 (1.01–1.45)	1.13 (0.95–1.36)	1.00	1.08 (0.92–1.27)
Multivariable adjusted HR	1.26 (1.08–1.54)	1.22 (1.01–1.46)	1.16 (0.97–1.38)	1.00	1.07 (0.91–1.27)
CVD mortality					
Baseline value					
No. of participants	1890	1879	1885	1878	1881
No. of cases	232	115	62	61	118
Person-years	9557	9813	10,359	10,489	10,320
Age- and sex-adjusted HR	3.19 (2.40–4.24)	1.89 (1.38–2.58)	1.01 (0.71–1.44)	1.00	1.98 (1.45–2.70)
Multivariable adjusted HR	3.52 (2.63–4.71)	2.00 (1.46–2.73)	1.03 (0.72–1.47)	1.00	1.67 (1.21–2.31)
Mean value					
No. of participants	1884	1883	1884	1882	1880
No. of cases	146	119	118	107	98
Person-years	9881	10,204	10,316	10,058	10,079
Age- and sex-adjusted HR	1.57 (1.17–1.10)	1.24 (0.92–1.68)	1.23 (0.90–1.69)	1.00	0.93 (0.70–1.22)
Multivariable adjusted HR	1.64 (1.22–2.21)	1.28 (0.94–1.74)	1.27 (0.92–1.74)	1.00	0.92 (0.70–1.22)

Data are hazard ratios (95% confidence intervals) unless otherwise indicated. Multivariable adjusted models included age, sex, body mass index, systolic blood pressure, HbA1c, low-density lipoprotein cholesterol, high-density lipoprotein cholesterol, triglycerides, estimated GFR, 25(OH)D, smoking status, use of antihypertensive drugs, use of glucose-lowering drugs, use of lipid-lowering drugs and use of antiplatelet or anticoagulant drugs

### CVD mortality

A total of 588 patients died from CVD during the follow-up. Multivariable-adjusted HRs of CVD mortality across quintiles of serum osteocalcin at baseline were 3.52 (95% CI 2.63–4.71), 2.00 (95% CI 1.46–2.73), 1.03 (95% CI 0.72–1.47), 1.00 and 1.67 (95% CI 1.21–2.31), respectively (Table 2). A significant U-shaped association of serum osteocalcin as a continuous variable with the risk of CVD mortality was observed when the restricted cubic spline curve was applied (Fig. 2B). The lowest hazards of CVD mortality occurred when serum osteocalcin levels at baseline were considered as 14.09 ng/mL and 24.16 ng/mL, compromising the optimal range from 14.22 to 24.42 ng/mL. When we used the updated mean values of serum osteocalcin as the exposures, the U-shaped association was also slightly attenuated to an L-shaped curve (Fig. 2D). The corresponding HRs of CVD mortality across quintiles of the updated mean of serum osteocalcin were 1.64 (95% CI 1.22–2.21), 1.28 (95% CI 0.94–1.74), 1.27 (95% CI 0.92–1.74), 1.00 and 0.92 (95% CI 0.70–1.22), respectively. The lowest hazards occurred when the mean osteocalcin level was considered as 15.56 ng/mL.

### Secondary analysis of the trajectories of osteocalcin during follow-up

In the present study, trajectories of osteocalcin were identified by using the latent class trajectory modeling as decreasing (Group 1), stable (Group 2), and increasing (Group 3) (Additional file 1: Fig. S1). When compared with patients with decreasing levels of osteocalcin during the follow-up, patients with stable or even increasing levels of osteocalcin had lower risks of all-cause mortality (HRs 0.48 (95% CI 0.40–0.58), and 0.50 (95% CI 0.36–0.70), respectively; Additional file 2: Table S1) and CVD mortality (HRs 0.55 (95% CI 0.40–0.76), 0.55 (95% CI 0.31–0.96), respectively).

### Subgroup analysis

When stratified analyses were utilized, the U-shaped association between baseline osteocalcin levels and the risks of all-cause mortality was consistent among patients of different ages, sexes, BMI, baseline HbA1c levels, renal functions, smoking status, and patients with and without using antiplatelet or anticoagulant, lipid-lowering, antihypertensive and glucose-lowering medications (Additional file 2: Table S2).

## Discussion

In this EHR-based cohort study, we demonstrated a U-shaped association between serum osteocalcin levels at baseline and the risks of all-cause and CVD mortality among patients with type 2 diabetes. Patients with lower levels of serum osteocalcin had higher risks for all-cause and CVD mortality. Subgroup analyses confirmed these consistent findings of patients with different baseline characteristics.

Osteocalcin is a polypeptide consisting of 46 to 50 amino acids [19], which is one of the specific markers of bone metabolism. Clinical measurements of serum osteocalcin can reflect bone turnover, bone renewal, and the functional role of osteoblasts. In recent years, the regulation of osteocalcin on glucolipid metabolism has been warmly discussed. However, most of the previous studies demonstrated a graded inverse linear association between serum osteocalcin and metabolic diseases and related comorbidities [20–22]. Only one study among community-dwelling older men in Australia demonstrated a U-shaped association between serum osteocalcin and the risks of all-cause and CVD-related mortality, which indicated that an optimal range for osteocalcin might exist to identify better health outcomes [23]. In the current study of real-world data, we found a U-shaped association between serum osteocalcin levels at baseline and the risk of all-cause and CVD mortality among patients with type 2 diabetes, which may provide solid evidence out of the limited studies worldwide. The optimal range associated with the lowest risk of mortality corresponds to around 14 ng/mL to 24 ng/mL. Notably, most of the traditional risk factors associated with mortality such as age, BMI, cholesterol panels, systolic blood pressure, and HbA1c at baseline across quintiles of osteocalcin were shown to be linear with different slopes. All these factors were already considered as covariates and adjusted in the models and we still found a strong U-shaped association between osteocalcin and mortality. The regulation of osteocalcin on metabolism may be bidirectional and more studies are recommended for the potential mechanism underlying this association.

A cluster of cytokines and adipokines have been involved in the interaction between osteocalcin and metabolic disorders such as adiponectin [24], adropin [25], leptin [26], and perilipin [27], etc. Previous studies have shown that osteocalcin was associated with multiple cardiometabolic risk factors such as body fat distribution [5], triglycerides [3], free fatty acid [28], hyperglycemia [3], and insulin resistance [29]. Osteocalcin was also associated with the presence of non-alcoholic fatty liver disease via activation of Nrf2 and inhibition of JNK [9]. Our team has investigated the association between osteocalcin and cardiovascular outcomes for decades. We found that serum total osteocalcin levels were associated with major adverse CVD events among patients at high risk of CVD. The osteocalcin levels might change during follow-up among patients with type 2 diabetes. Multiple measurements of osteocalcin also allow us to give a comprehensive insight into the associations. Interestingly, we observe an L-shaped association between serum osteocalcin levels and mortality (both all-cause and CVD mortality) when the updated mean values of serum osteocalcin were the exposure. Simultaneously, patients with a decreasing trend of serum osteocalcin levels had a higher risk of mortality than those in other groups. The incidence of all-cause mortality in patients with a decreasing trend of serum osteocalcin levels was 36.2%, which was much higher than those in other groups. This decline of osteocalcin levels from baseline to follow-ups may weaken the U-shaped association at baseline, which also supported the findings that lower osteocalcin levels were associated with higher risks of mortality.

The major cause of death in the present cohort is cancer (about one-third). Osteocalcin was shown to be a potential biomarker for metastatic malignancies such as breast cancer [30], prostate cancer [31], hepatitis B virus-related hepatocellular carcinoma [32], or other bone-derived tumors. The underlying mechanism involves the cAMP-dependent protein kinase A signaling pathway [33], cyclo-oxygenase-2 signaling pathway [34], osteoblast-specific expression of Fra-2/AP-1 pathway [35], etc. Longitudinal evidence in terms of this topic is lacking, especially among patients with type 2 diabetes. A recent study in patients with hemodialysis suggested a potential protective role of osteocalcin in the bone, endocrine, and vascular pathways [36]. Osteocalcin also predicted all-cause mortality in postmenopausal women [37]. In this study, we have provided new real-world-based evidence for this association. However, whether exogenous administration of osteocalcin can improve longevity is still a question for future research.

The major strength of this study is the large sample size, which allows us to have sufficient statistical power to conclude a certain non-linear association between osteocalcin and mortality among patients with type 2 diabetes. In addition to a single measurement of osteocalcin, we also performed a trajectory analysis including three consecutive measurements of osteocalcin. When compared with patients who had a decreasing tendency of osteocalcin, those with a stable level of osteocalcin and with an increasing tendency of osteocalcin had a lower hazard of either all-cause mortality or CVD mortality. On the other hand, a stable or increasing level of osteocalcin may contribute to a longer life span, but it is not the higher the better. Other strengths of this study include the long follow-ups and the relatively rich data in real-world clinical practice. Some limitations need to be addressed. First, the measurement of osteocalcin is not a routine check-up in the outpatient clinic so only patients who initiated inpatient visits in our center were included, which may cause selection bias. In addition, some socioeconomic variables were missing in the EMR data including education level, family income, etc. We only measured total osteocalcin levels in this study, which may limit us to distinguish uncarboxylated osteocalcin from the total one. Uncarboxylated osteocalcin is the biologically active isoform mediating the metabolic functions and may have different prognostic significance than the total one. However, several studies have demonstrated that not only undercarboxylated but also carboxylated osteocalcin and total ones were associated with glucose and fat metabolism as well [3, 38, 39]. We will validate whether undercarboxylated osteocalcin or uncarboxylated osteocalcin is also associated with all-cause and CVD mortality or not in our future studies. Finally, the data used in this analysis were from a single center in Shanghai, where some sociodemographic characteristics of healthcare system members may differ from those in other geographic areas in China. The generalizability of our findings was thus limited.

## Conclusions

In conclusion, the present analysis using data from the Shanghai Clinical Center for Diabetes showed a U-shape association between serum osteocalcin levels and the risks of all-cause and CVD mortality among patients with type 2 diabetes. Secondary trajectory analyses also supported that patients with lower levels of serum osteocalcin had higher risks for all-cause and CVD mortality. Further interventional trials are challenging in targeting the treatment effect of osteocalcin.

## Supplementary Information

**Additional file 1: Figure S1. **Trajectories of osteocalcin levels during follow-ups. The red line stands for patients with decreasing osteocalcin levels during follow-ups. The blue line stands for patients with stable osteocalcin levels during follow-ups. The yellow line stands for patients with increasing osteocalcin levels during follow-ups.

**Additional file 2: Table S1.** Hazard ratios for all-cause mortality and CVD mortality per trajectory of osteocalcin. **Table S2.** Subgroup analysis according to different baseline characteristics in association with all-cause mortality.

## Data Availability

The datasets generated and/or analyzed in the current study are not publicly available but are available from the corresponding author on reasonable request.

## Abbreviations

BMI: Body mass index
CI: Confidence interval
CKD-EPI: Chronic Kidney Disease Epidemiology Collaboration equation
COD: Confirmation of death
CVD: Cardiovascular disease
eGFR: Estimated glomerular filtration rate
EHR: Electronic health record
HbA1c: Glycated hemoglobin A1c
HDL: High-density lipoprotein
HR: Hazard ratios
ICD-10: International Classification of Disease 10th version
LDL: Low-density lipoprotein
25(OH)D: 25 Hydroxyvitamin D
