# Supplementary figures and images for: Low total osteocalcin levels are associated with all-cause and cardiovascular mortality among patients with type 2 diabetes: a real-world study
Source: Cardiovasc Diabetol. 2022 Jun 9;21:98. doi: 10.1186/s12933-022-01539-z (PMC9185881; doi:10.1186/s12933-022-01539-z)

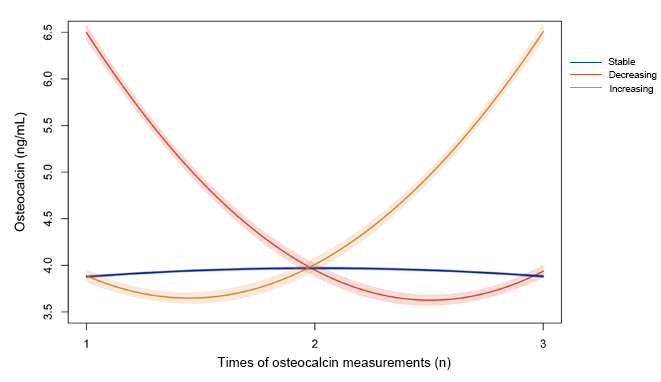

Supplement: Supplementary file 1 — Additional file 1: Figure S1. Trajectories of osteocalcin levels during follow-ups. The red line stands for patients with decreasing osteocalcin levels during follow-ups. The blue line stands for patients with stable osteocalcin levels during follow-ups. The yellow line stands for patients with increasing osteocalcin levels during follow-ups. [file 12933_2022_1539_MOESM1_ESM.tif]
