# Supplementary material for: Low total osteocalcin levels are associated with all-cause and cardiovascular mortality among patients with type 2 diabetes: a real-world study
Source: Cardiovasc Diabetol. 2022 Jun 9;21:98. doi: 10.1186/s12933-022-01539-z (PMC9185881; doi:10.1186/s12933-022-01539-z)
Supplement: Supplementary file 2 — Additional file 2: Table S1. Hazard ratios for all-cause mortality and CVD mortality per trajectory of osteocalcin. Table S2. Subgroup analysis according to different baseline characteristics in association with all-cause mortality. [file 12933_2022_1539_MOESM2_ESM.docx]

Table S1. Hazard ratios for all-cause mortality and CVD mortality per trajectory of osteocalcin.

|  | Trajectories of osteocalcin | | |
| --- | --- | --- | --- |
|  | Decreasing  (Group 1) | Stable  (Group 2) | Increasing  (Group 3) |
| **All-cause mortality** |  |  |  |
| No. of participants | 354 | 8,711 | 348 |
| No. of cases | 128 | 1461 | 49 |
| Person-years | 1,866 | 46,889 | 1,784 |
| Age- and sex- adjusted HR | 1.00 | 0.47 (0.39-0.56) | 0.49 (0.35-0.68) |
| Multivariable adjusted HR | 1.00 | 0.48 (0.40-0.58) | 0.50 (0.36-0.70) |
| **CVD mortality** |  |  |  |
| No. of participants | 354 | 8,711 | 348 |
| No. of cases | 35 | 536 | 17 |
| Person-years | 1,866 | 46,889 | 1,784 |
| Age- and sex- adjusted HR | 1.00 | 0.54 (0.39-0.74) | 0.54 (0.31-0.94) |
| Multivariable adjusted HR | 1.00 | 0.55 (0.40-0.76) | 0.55 (0.31-0.96) |

Data are hazard ratios (95% confidence intervals) unless otherwise indicated. Multivariable adjusted models included age, sex, body mass index, systolic blood pressure, HbA1c, low-density lipoprotein cholesterol, high-density lipoprotein cholesterol, triglycerides, estimated GFR, 25(OH)D, smoking status, use of antihypertensive drugs, use of glucose-lowering drugs, use of lipid-lowering drugs and use of antiplatelet or anticoagulant drugs.

Table S2. Subgroup analysis according to different baseline characteristics in association with all-cause mortality.

|  | Baseline osteocalcin (ng/mL) | | | | |
| --- | --- | --- | --- | --- | --- |
|  | Quintile 1 | Quintile 2 | Quintile 3 | Quintile 4 | Quintile 5 |
| Age, years old |  |  |  |  |  |
| <65 | 3.56 (2.70-4.69) | 1.79 (1.33-2.41) | 1.16 (0.84-2.41) | 1.00 | 2.49 (1.92-3.23) |
| ≥65 | 2.90 (2.33-3.61) | 1.72 (1.36-2.19) | 1.26 (0.97-1.62) | 1.00 | 1.33 (1.06-1.67) |
| Sex |  |  |  |  |  |
| Male | 2.79 (2.21-3.50) | 1.70 (1.32-2.18) | 1.22 (0.94-1.59) | 1.00 | 1.99 (1.60-2.47) |
| Female | 3.33 (2.56-4.32) | 1.70 (1.27-2.28) | 1.15 (0.84-1.57) | 1.00 | 1.77 (1.35-2.32) |
| Body mass index, kg/m^2^ |  |  |  |  |  |
| <25 | 2.86 (2.31-3.55) | 1.81 (1.44-2.28) | 1.24 (0.97-1.58) | 1.00 | 1.81 (1.48-2.22) |
| 25-29.9 | 2.83 (2.06-3.87) | 1.52 (1.07-2.17) | 1.03 (0.70-1.51) | 1.00 | 1.99 (1.43-2.77) |
| ≥30 | 3.65 (1.75-7.63) | 1.03 (0.44-2.44) | 1.16 (0.48-2.83) | 1.00 | 1.49 (0.68-3.26) |
| HbA1c, % |  |  |  |  |  |
| <7.0 | 2.91 (2.07-4.09) | 1.52 (1.05-2.21) | 1.30 (0.89-1.91) | 1.00 | 1.93 (1.44-2.61) |
| ≥7.0 | 2.90 (2.37-3.54) | 1.72 (1.38-2.14) | 1.15 (0.91-1.45) | 1.00 | 1.57 (1.27-1.94) |
| Estimated GFR, mL/min/1.73 m^2^ |  |  |  |  |  |
| <60 | 5.16 (2.27-11.7) | 3.59 (1.52-8.48) | 2.12 (0.90-5.01) | 1.00 | 1.06 (0.62-1.80) |
| ≥60 | 2.74 (2.30-3.27) | 1.56 (1.28-1.89) | 1.11 (0.90-1.36) | 1.00 | 1.74 (1.45-2.08) |
| Smoking status |  |  |  |  |  |
| Current and past smoking | 3.39 (2.36-4.88) | 1.64 (1.11-2.42) | 1.35 (0.91-2.01) | 1.00 | 2.03 (1.66-2.48) |
| Never smoking | 2.74 (2.26-3.34) | 1.66 (1.34-2.06) | 1.10 (0.87-1.39) | 1.00 | 1.31 (0.93-1.85) |
| Antiplatelet or anticoagulant |  |  |  |  |  |
| No use | 3.71 (2.56-3.69) | 1.79 (1.47-2.18) | 1.13 (0.91-1.41) | 1.00 | 1.80 (1.49-2.17) |
| Use | 1.67 (1.01-2.77) | 1.23 (0.73-2.08) | 1.01 (0.47-1.61) | 1.00 | 1.84 (1.26-2.68) |
| Lipid-lowering medications |  |  |  |  |  |
| No use | 3.05 (2.39-3.88) | 1.73 (1.34-2.25) | 1.25 (0.95-1.64) | 1.00 | 1.72 (1.37-2.17) |
| Use | 2.64 (2.06-3.39) | 1.53 (1.17-2.01) | 1.08 (0.81-1.47) | 1.00 | 2.09 (1.63-2.69) |
| Antihypertensive medications^‡^ |  |  |  |  |  |
| No use | 2.75 (1.67-4.50) | 2.10 (1.26-3.51) | 1.16 (0.62-2.15) | 1.00 | 2.00 (1.19-3.38) |
| Use | 2.95 (2.45-3.55) | 1.56 (1.27-1.91) | 1.16 (0.94-1.44) | 1.00 | 1.85 (1.54-2.21) |
| Glucose-lowering medications |  |  |  |  |  |
| No use | 3.79 (2.76-5.22) | 1.93 (1.37-2.74) | 0.96 (0.64-1.44) | 1.00 | 1.66 (1.14-2.42) |
| Metformin | 2.55 (2.06-3.15) | 1.61 (1.28-2.01) | 1.27 (1.01-1.60) | 1.00 | 1.82 (1.51-2.20) |
| Insulin | 2.08 (1.62-2.68) | 1.36 (1.04-1.77) | 1.11 (0.85-1.46) | 1.00 | 1.90 (1.52-2.36) |
| Insulin secretagogues | 1.77 (1.19-2.65) | 1.04 (0.77-1.42) | 1.16 (0.92-1.46) | 1.00 | 2.26 (1.27-4.02) |
| DPP4 inhibitors | 1.69 (1.20-2.38) | 1.03 (0.72-1.47) | 0.87 (0.57-1.32) | 1.00 | 1.32 (0.64-2.73) |
| α-glucosidase inhibitors | 2.75 (2.25-3.36) | 1.65 (1.33-2.06) | 1.19 (0.94-1.50) | 1.00 | 1.89 (1.56-2.30) |
| GLP-1 receptor agonists | 8.62 (1.22-61.1) | 0.99 (0.22-4.56) | 1.03 (0.30-3.57) | 1.00 | 9.45 (1.91-46.8) |
| Thiazolidinediones | 1.88 (1.40-2.54) | 1.11 (0.80-1.54) | 1.03 (0.75-1.42) | 1.00 | 1.88 (1.45-2.45) |
| SGLT2 inhibitors^‡^ | 3.42 (0.10-132) | 0.53 (0.01-49.7) | 0.38 (0.10-28.9) | 1.00 | 10.5 (1.33-83.2) |

Data are hazard ratios (95% confidence intervals) unless otherwise indicated. Multivariable adjusted models included age, sex, body mass index, systolic blood pressure, HbA1c, low-density lipoprotein cholesterol, high-density lipoprotein cholesterol, triglycerides, estimated GFR, smoking, use of antiplatelet or anticoagulant drugs, use of lipid-lowering drugs, use of antihypertensive drugs, and use of glucose-lowering drugs, other than variables for stratification.
